# The immunological effect of Galectin-9/TIM-3 pathway after low dose Mifepristone treatment in mice at 14.5 day of pregnancy

**DOI:** 10.1371/journal.pone.0194870

**Published:** 2018-03-22

**Authors:** Adrienn Lajko, Matyas Meggyes, Beata Polgar, Laszlo Szereday

**Affiliations:** 1 University of Pecs, Medical School, Department of Medical Microbiology and Immunology, Pecs, Hungary; 2 Janos Szentagothai Research Centre, Pecs, Hungary; Xavier Bichat Medical School, INSERM-CNRS - Université Paris Diderot, FRANCE

## Abstract

The abortifacient Mifepristone (RU486) has proven to be a safe, effective, acceptable option for millions of women seeking abortion during the first and second trimester of pregnancy although its precise mechanism of action is not well understood. The main objective of this study was to investigate the impact of low dose Mifepristone administration on placental Galectin-9 (Gal-9) expression, as well as its effect on the cell surface expression of Gal-9, TIM-3 and CD107a molecules by different T and NK cell subsets. A model of Mifepristone-induced immunological changes was established in syngeneic pregnant BALB/c mice. RU486-induced alteration in placental Gal-9 expression was determined by immunohistochemistry. For immunophenotypic analysis, mid-pregnancy decidual lymphocytes and peripheral mononuclear cells were obtained from Mifepristone treated and control mice at the 14.5 day of gestation. TIM-3 and Gal-9 expression by peripheral and decidual immune cells were examined by flow cytometry. Our results revealed a dramatically decreased intracellular Gal-9 expression in the spongiotrophoblast layer of the haemochorial placenta in Mifepristone treated pregnant mice. Although low dose RU486 treatment did not cause considerable change in the phenotypic distribution of decidual and peripheral immune cells, it altered the Gal-9 and TIM-3 expression by different NK and T cell subsets. In addition, the treatment significantly decreased the CD107a expression by decidual TIM-3+ NK cells, but increased its expression by decidual NKT cell compared to the peripheral counterparts. These findings suggest that low dose Mifepristone administration might induce immune alterations in both progesterone dependent and independent way.

## Introduction

Unintended pregnancy is a major globe tragedy for millions of women representing significant direct and indirect costs to health care, no matter for individuals or society. The World Health Organization (WHO) estimates that approximately 40–60 million abortions were induced worldwide each year [1]. During the first and second trimester, medical or surgical abortion is one of the oldest, most commonly practiced and most controversial procedure performed worldwide.

Since its approval in France in 1988, the abortifacient Mifepristone (RU486) has proven to be a safe, effective, acceptable option for millions of women seeking abortion during the first several weeks of pregnancy [2]. Mifepristone also proved to be a safe and effective method for pregnancy termination during the second-trimester (mainly between the 13 and 20 weeks) with a combination of the synthetic prostaglandin E1 analog Misoprostol [3,4]. Second-trimester medical abortions constitute 10–15% of all induced abortions worldwide [3]. Administration of Mifepristone followed by prostaglandin and misoprostol has been used successfully in the medical termination of pregnancy for over 27 years, and the method is registered in 50 countries [5]. Although it is well tolerated, there still remain a few adverse reactions and side effects, like abdominal pain, nausea, vomiting and diarrhea, and it may also cause complications of hemorrhage and sepsis.

Until now, the exact mechanism of action of Mifepristone is not well investigated and has to be fully elucidated, therefore the development of an animal model that captures the effects of Mifepristone-induced immunological changes during pregnancy may help to expand our understanding of the biological and cellular basis of the abortion process.

Previous data reported that RU486 significantly reduced the quantity and function of Treg cells in the fetal-maternal interface before the onset of induced abortion [6]. Li et al. demonstrated that RU486 blocked the Th2 and Treg predominance in pregnancy and promoted Th1 and Th17 skews in mice at the maternal–fetal interface, which contributes to the termination of pregnancy [7]. Similar findings show a high level of Th1 type cytokines (IFN-γ, IL-2) was found in RU486-induced abortion mice, while the Th2 type cytokine (IL-4, IL-10) levels were not influenced significantly [8]. Bogdan et al. showed that in decidual NK cells perforin co-localizes with PIBF in the granules, and that while in RU486-treated mice (0.8 mg/kg) PIBF positive NK cell counts decreased by approximately 50%, the ratio of perforin positive cells increased within the PIBF positive population [9].

Several previous research studies have established that Gal-9/Tim-3 pathway play an essential role in immunoregulation and induction of tolerance [10–13]. TIM-3 expression was verified in a variety of immune cells, including Th1, Th17, NK and NKT cells, Tregs, and also on antigen presenting immune cells [14]. TIM-3 molecule has been implicated in both activation and inhibition of immune response [15,16]. It was presented that expression of TIM-3 on Th1 cells provides a key checkpoint that serves to dampen proinflammatory Th1-dependent T-cell response and may contribute to the maintenance of healthy pregnancy, but it’s *in vivo* expression and function after Mifepristone treatment is still unknown [12].

Among the several identified receptors of Gal-9, TIM-3 has been studied most extensively. There are evidence that engagement of TIM-3 by its ligand Gal-9 leads to the apoptosis of Th1 and Th17 cells and induce T cell tolerance both in mice and humans [17–19]. Thus, engagement of TIM-3 by Gal-9 may function as a negative immune-regulator abrogating the Th1- and Th17-driven immune response and therefore modulate the Th1/Th2 cytokine balance. In this regard, it is supposed that Gal-9/TIM-3 interaction could play an important role in the regulation of maternal immune tolerance towards the fetus and may be a potent regulator of the innate and adaptive immune response. Since the influence of Mifepristone treatment on Gal-9/TIM-3 pathway is still unknown, exploring the relationship between Gal-9/TIM-3 pathway and Mifepristone-induced changes during pregnancy may provide a better understanding of the pathogenesis of immunological changes during immune-mediated abortions.

In the present study, we examined the alterations of the Gal-9/TIM-3 pathway that might play an important role in the immunological changes caused by Mifepristone treatment. The aim of our study was to characterize the Mifepristone-induced changes on the placental Gal-9 production and the immunological alterations at the materno-fetal interface and in the periphery by using a syngeneic pregnant mouse model.

## Material and methods

### Animal model

BALB/c mice were provided by the Experimental Central Animal Laboratory of the University of Pecs. Animal housing, care and application of experimental procedures were in accordance with institutional guidelines under approved protocols (No. BA02/ 2000-7/2015, University of Pecs). All animals were caged in a temperature- and light controlled environment with 12 hours light and 12 hours dark cycle, and received ad libitum water and food for maintenance. 2 month-old female mice were mated with fertile male mice overnight to establish pregnancy. Successful mating was confirmed by the presence of copulatory (vaginal) plug in the next morning and it was designated as the day 0.5 of pregnancy.

### Mifepristone (RU486) treatment

29 pregnant mice were assigned to the Mifepristone-treated group and 30 non-treated pregnant mice were selected as controls. In our experimental model, Mifepristone was applied at a dosage of 0.8 mg/kg to trigger immunological changes with the minimal impairment of the decidua. RU486 (Sigma-Aldrich) was applied on gestational day (gd) 14.5 by a single intraperitoneal injection at the dosage of 0.8 mg/kg body weight, freshly dissolved in 200μl phosphate-buffered saline (PBS) before use. In the normal pregnancy control group, mice received an intraperitoneal injection of 200μl sterile PBS on day 14.5 of pregnancy. Mifepristone treated and control pregnant females were killed by cervical dislocation next morning (on the gd 15.5), their spleen and the uterine horns were removed aseptically and were processed as indicated below.

### Immunohistochemistry

Placental tissue was removed from uterine horns and fixed with 4% paraffin for more than 24hours. Next, the samples were embedded in paraffin and 4μm thick sections were prepared. After deparaffinization in xylol for 3x5 minutes, samples were rehydrated in degraded alcohol series (96%, 80%, 70%, 50%) for 3 minutes each. Then tissue sections were washed in distilled water (dH2O) and antigen retrieval was performed for 20 minutes in boiling 1x Target Retrieval Solution (TRS) (1:10; pH 6.0–6.2; Dako). Next, the samples were cooled down to RT, washed twice with dH_2_O and endogenous peroxidase was blocked for 15 minutes in 3%H_2_O_2_. Then samples were washed 3x5 minutes with 50mM Tris buffer saline (TBS) supplemented with 0.05% Tween pH7.4 (TBST) and pre-blocked with 3%BSA (Sigma-Aldrich) for 20 minutes. Following washing with TBST for 2x5 minutes slides were incubated with biotinylated goat anti-mouse Gal-9 antibody (1:10; R&D Systems) for 1 hour at RT. After washing three times in TBST, samples were incubated with Streptavidin-Biotinylated Horseradish Peroxidase Complex (1:100; GE Healthcare) for 30 minutes at RT. Following the washing procedure, the signal was detected with Liquid DAB+Substrate Chromogen System (Dako) for 5 to 30 minutes at RT. Hematoxylin counterstain was performed for 3 minutes at RT then slides were covered with mounting medium (Dako).

### Isolation of mononuclear cells from the spleen

Spleens were homogenized thoroughly with a syringe plunger, and single-cell suspensions were prepared using a 70μm nylon cell strainer (BD-Biosciences). Subsequently, cells were washed in PBS. The supernatant was aspirated and the pellet was resuspended in PBS and filtered again via 40μm nylon cell strainer (BD-Biosciences). Then, mononuclear cells were separated by Ficoll-Paque Premium 1.084 gradient (GE Healthcare). Isolated cells were collected and resuspended in RPMI1640 (Lonza) medium supplemented with penicillin (1x10^5^U⁄L, Lonza), streptomycin (0.05g⁄L, Lonza) and 10% fetal bovine serum (FBS)(Gibco).

### Isolation of mononuclear cells from the decidua

Decidual mononuclear cells from the materno-fetal interface were isolated according to the method described previously [20]. Briefly, the endometrial tissue surrounding the conceptuses was pulled away from the uterine horns. Than the decidua was separated from the placenta disc under a dissecting microscope, sliced with scissors and digested with type IV collagenase (Sigma-Aldrich) in 37°C for 30 minutes. The suspensions were homogenized with a syringe plunger, and single-cell suspensions were prepared using a 70μm nylon cell strainer. Subsequently, cells were washed in RPMI1640 medium supplemented with penicillin, streptomycin and 10% FBS. The supernatant was aspirated, the pellet was resuspended in PBS and filtered via 40μm nylon cell strainer. Finally, the isolated cells were resuspended in RPMI1640 + 10% FBS.

### Labeling of lymphocytes and flow cytometric analysis

For surface labeling isolated lymphocytes (10^6^cells/100μl PBS/tube) were incubated for 30 min at room temperature (RT) with the fluorochrome-labelled monoclonal antibodies indicated below. After washing with PBS cells were resuspended in 300μl PBS containing 1% paraformaldehyde and stored at 4°C in the dark until fluorescence-activated cell sorting (FACS) analysis. Before sample analysis, the setting of the flow cytometer was checked using Cytometer Setup and Tracking beads (CS&T beads, BD-Biosciences) according to the manufacturer’s instructions. Compensation beads were used with single stains of each antibody in order to determine the compensation settings, and were applied in FACSDivaTM V6 software (BD-Biosciences) before data collection. Labeled cells were analyzed with FACSCantoII^TM^ flow cytometer by collecting 100,000 events in the lymphogate in the case of the spleen. In the case of decidua 10,000 events were collected in the lymphogate after CD45 staining. FACSDiva^TM^ V6 software was used for data acquisition and analysis.

### Antibodies

The following monoclonal antibodies were used during the assay: Brilliant violet (BV)510-conjugated anti-mouse CD3 (BD-Biosciences), BV510-conjugated anti-mouse γ/δTCR (BD-Biosciences) recognizing delta chain, fluorescein isothiocyanate (FITC)-conjugated anti-mouse CD4 (BD-Biosciences), FITC-conjugated anti-mouse CD107a (BD-Biosciences), FITC-conjugated anti-mouse CD49b (BD-Biosciences), phycoerythrin (PE)-conjugated anti-mouse TIM-3 (R&D Systems), PE-conjugated anti-mouse Gal-9 (Biolegend), PE-conjugated anti-mouse CD49b (BD-Biosciences), peridinin chlorophyll a protein (PerCP)-conjugated anti-mouse CD45 (Exbio), PE-Cy7-conjugated anti-mouse CD25 (BD-Biosciences), allophycocyanin (APC)-conjugated anti-mouse TIM-3 (R&D Systems), APC-conjugated anti-mouse FoxP3 (eBioscience) and APC-H7-conjugated anti-mouse CD8 (BD-Biosciences). Control antibodies included isotype-matched rat and hamster antibodies.

### FoxP3 staining

Following surface labeling, intracellular staining of FoxP3 was performed using the FoxP3 Staining Buffer Set (eBioscience) in accordance with the manufacture’s protocol. Briefly, isolated cells were permeabilized in 1ml fixation/permeabilization buffer (Concentrate/Diluent 1:4 eBioscience) for 1 hour at 4°C. Then the samples were washed twice in the buffer and stained with APC-conjugated anti-mouse FoxP3 monoclonal antibody for 1 hour at 4°C. FACS analysis was performed by FACSCantoII^TM^ flow cytometer with the BD FACSDiva^TM^ software for data acquisition and analysis.

### Detection of CD107a expression

The CD107a assay was set up based on a publication by Alter et al. [21]. To determine CD107a expression by immune cells, mononuclear cells were incubated for 4h at 37°C with FITC-conjugated anti-mouse CD107a antibody in RPMI1640 containing 10% FBS, penicillin, streptomycin, ionomycin (Sigma-Aldrich) and phorbol myristate acetate (PMA) (Sigma-Aldrich). Next, the cells were washed with PBS and incubated with anti-CD3-BV510, anti-γ/δ-BV510, anti-CD49b-PE, anti-CD45-PerCP and anti-TIM-3-APC antibodies for 30 minutes at RT in the dark. Then the cells were washed in PBS, fixed with 1% paraformaldehyde (PFA) and evaluated by FACS.

### Quantification of immunohistochemistry

The stained slides were evaluated by two independent researchers, who were blinded to treatment groups. Sections stained by immunohistochemistry were analyzed for the percentages of Gal-9 positive cells. During analysis, a total of 100 cells/slide were evaluated at 350× magnification.

### Statistical analysis

Statistical analysis was performed using statistical software SPSS version 20 package. Multiple comparisons were made using one-way ANOVA with Bonferroni correction. Differences were considered significant if the p value was equal to or less than 0.05.

## Results

### Immunohistochemistry of pregnant mouse placentae from untreated control and RU486 treated pregnant mice

**Fig 1:**
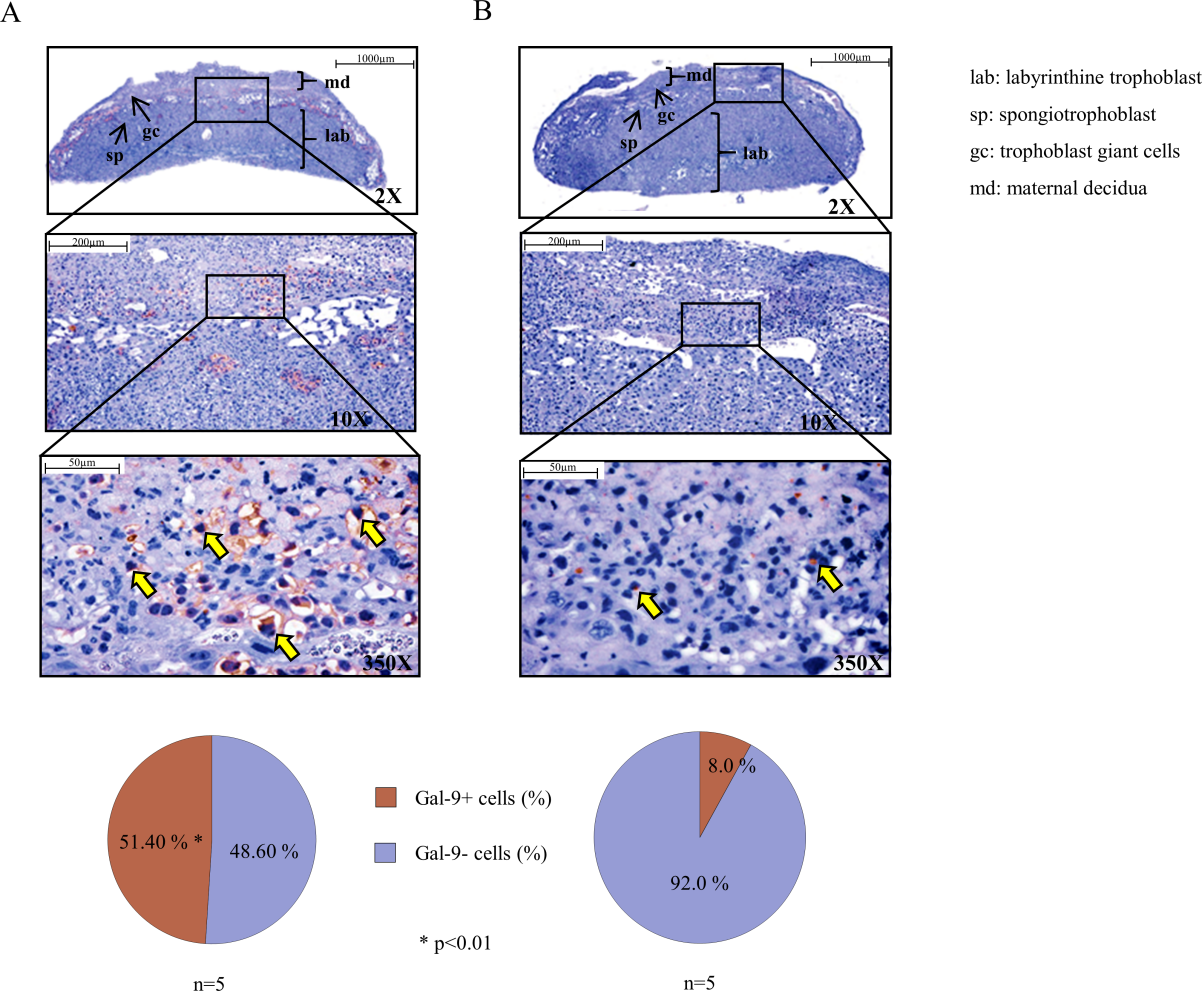
Representative Gal-9 immunohistochemical staining of pregnant mouse placentae from untreated control and RU486 treated pregnant mice. IHC was performed on the placenta-sections of healthy control (Fig 1A) and RU486 treated (Fig 1B) mice. Yellow arrows indicate Gal-9 positive cells (magnification 350x). The lower circle graphs show the percentage of cytoplasmic Gal-9 positive cells (data are shown as mean). Differences were considered statistically significant for p-values ≤0.05. The images were captures utilizing the Pannoramic DESK scanner (3DHISTECH Ltd.) and analysis was performed by the Pannoramic viewer software (3DHISTECH Ltd.).

Immunohistochemistry on isolated healthy pregnant mouse placentae with anti-Gal-9 antibody demonstrated a strong expression of Gal-9 (yellow arrow) at the spongiotrophoblast layer and in the cytoplasm of giant cells (Fig 1A). Histological sections represented a more intense staining by trophoblast giant cells compared to spongiotrophoblast layer (Fig 1A). Evaluation of the number of positively stained cells showed a significant decrease of Gal-9 positive cells in the placentae of RU486 treated pregnant mice compared to the placentae of healthy, non-treated pregnant mice (Fig 1B).

### Immunophenotypic analysis of decidual and peripheral mononuclear cells from untreated control and RU486 treated pregnant mice

In our study, we investigated the percentage of CD4+T, CD8+T, γ/δT, Treg, NK and NKT cells using the lymphocyte gate in the spleen (periphery) and in the decidua (locally) of untreated and RU486 treated pregnant mice.

RU486 treatment did not cause a statistically significant change in the phenotype distribution of immune cells both in the decidua and in the periphery when compared to untreated mice (Table 1). Albeit previous literature data presented contradictory results about the impact of RU486 on the distribution of decidual NK cells [22][23] our results were in accordance with the earlier human immunohistochemistry results by Milne et al. [24] who found that simultaneous administration of Mifepristone and the prostaglandin E analog did not affect the number of NK cells in the decidua.

**Table 1:** Phenotype analysis of decidual and peripheral mononuclear cells from untreated control and RU486 treated pregnant mice.

	Periphery (n = 12)		Decidua (n = 12)	
	Untreated mice	Mifepristone treated mice	Untreated mice	Mifepristone treated mice
**CD4+ T cells**	35.90±2.06	40.70±1.85	10.00±1.50	8.36±1.30
**CD8+ T cells**	13.61±0.89	13.43±0.88	4.85±0.72	3.46±0.92
**Treg cells**	2.67±0.23	2.71±0.36	0.39±0.05	1.07±0.28
γ/δ**T cells**	4.57±0.56	3.78±0.48	14.56±2,93	16.55±2.61
**NKT cells**	1.76±0.16	1.80±0.26	14.38±1.34	17.77±4.00
**NK cells**	12.36±0.51	11.14±0.85	28.16±1.37	24.77±1.18

Multiple comparisons were made using one-way ANOVA with Bonferroni correction. The results were expressed as the mean value±standard error of the mean (SEM). Differences were considered significant when the value of p was equal to or less than 0.05.

### Cell surface Gal-9 expression by decidual and peripheral mononuclear cells isolated from untreated control and RU486 treated pregnant mice

Flow cytometry was used to analyze the cell surface expression of Gal-9 by NK, NKT, γ/δT, CD4+T and Treg cells at the materno-fetal interface as well as in the periphery.

**Fig 2:**
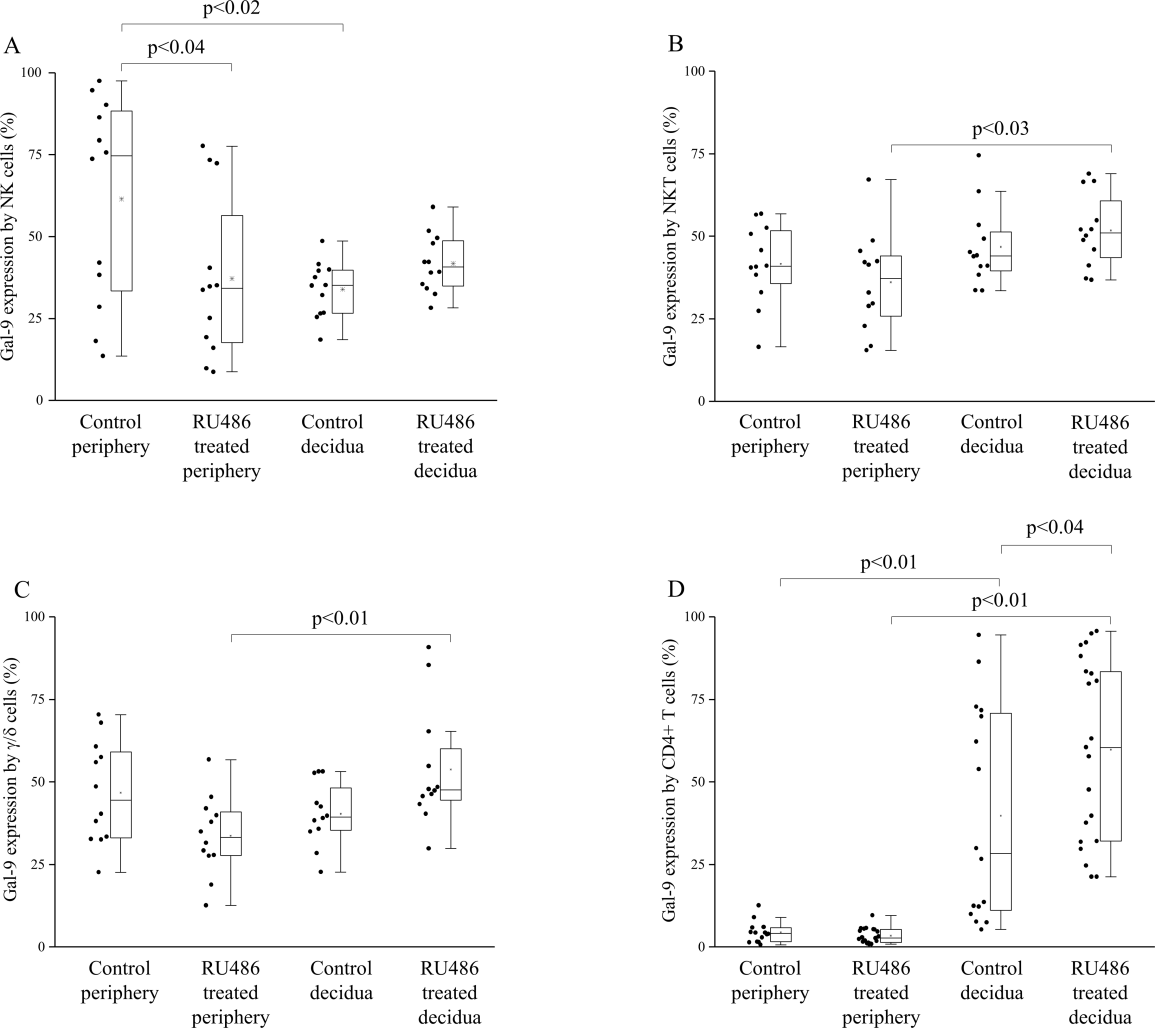
Gal-9 expression by decidual and peripheral mononuclear cells from untreated control and RU486 treated pregnant mice. Box plot of the median, the 25th and 75th percentiles, range, and individual data values for the cell surface expression of Gal-9 by NK cells, NKT cells, γ/δT, CD4+ T cells in periphery and decidua of untreated and RU486 treated pregnant mice. The middle line within the box represents the median, the middle dot within the box represents the mean, the boxes indicate the interquartile ranges and the whiskers show the most extreme observations. Differences were considered statistically significant for p-values ≤0.05.

**Fig 3:**
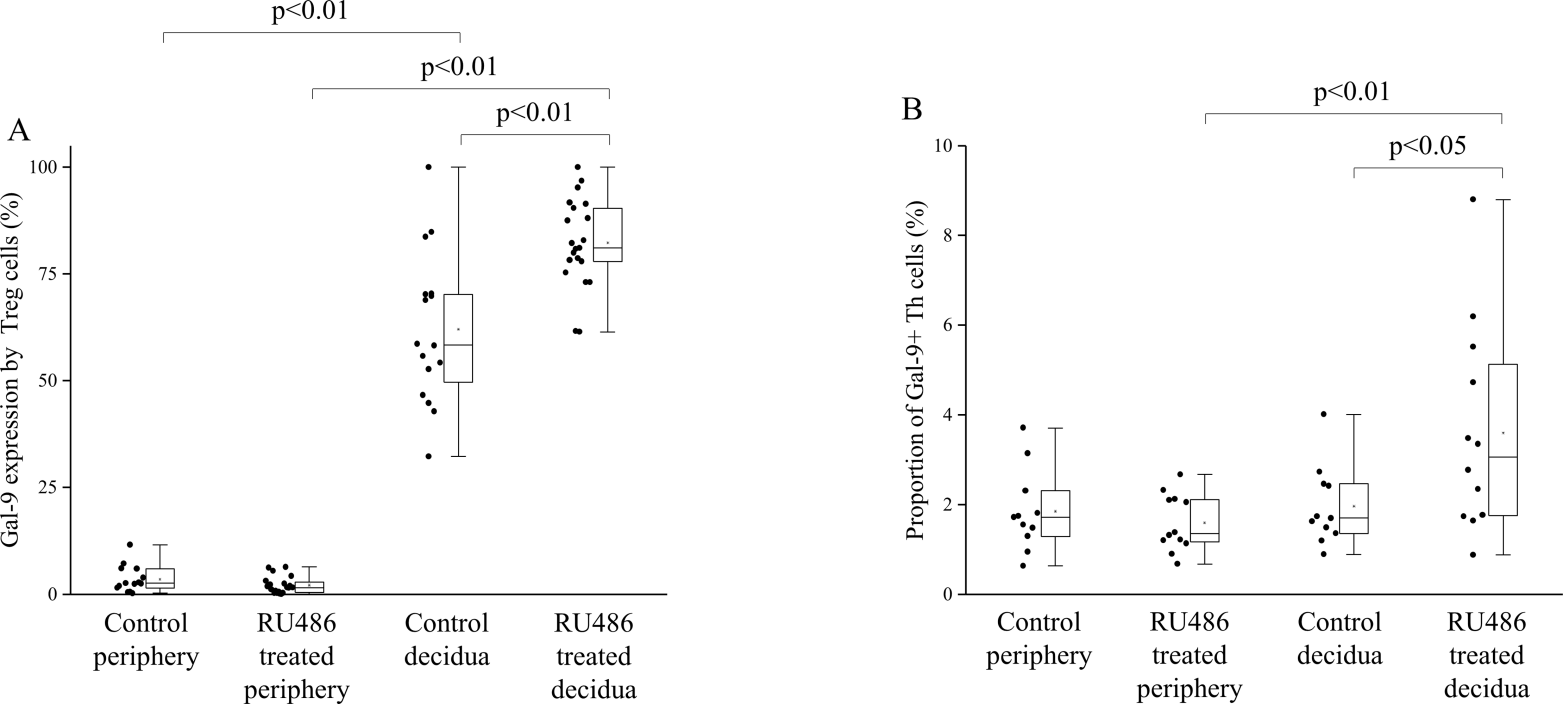
Gal-9 expression by Tregs and the proportion of Gal-9+ Th cell population in decidua and periphery from untreated control and RU486 treated pregnant mice. Box plot of the median, the 25th and 75th percentiles, range, and individual data values for the cell surface expression of Gal-9 by Treg cells and the frequency of Gal-9 positive Th cells in decidua and periphery from untreated control and RU486 treated pregnant mice. The middle line within the box represents the median, the middle dot within the box represents the mean, the boxes indicate the interquartile ranges and the whiskers show the most extreme observations. Differences were considered statistically significant for p-values ≤0.05.

In untreated pregnant mice Gal-9 expression by decidual Treg and CD4+T cells was significantly increased (Figs 2D and 3A), while decidual NK cells showed a significantly decreased Gal-9 expression compared to peripheral NK cells (Fig 2A). Gal-9 positivity of decidual NKT and γ/δ T cells showed no significant difference compared to their peripheral counterparts (Fig 2B and 2C). In RU486 treated mice Gal-9 expression was significantly increased by almost all investigated decidual subpopulations when compared to the periphery except NK cells (Figs 2A, 2B, 2C, 2D and 3A). After RU486 administration Gal-9 expression by decidual Treg and CD4+T cells were significantly increased in comparison to the decidua of untreated control mice (Figs 2D and 3A), while its expression was significantly decreased in the periphery after RU486 treatment by NK cells compared to untreated mice (Fig 2A).

### The proportion of Gal-9+ T-helper cell population in decidua and periphery of untreated control and RU486 treated pregnant mice

It was published by Oomizu et al., that CD4+ T-helper (Th) lymphocytes expressing Gal-9 on their cell surface possess immunosuppressive potential by regulating the Th17/Treg balance [25]. In order to examine the impact of Mifepristone administration on the distribution of this T cell-subset, we investigated the percentage of Gal-9+ Th cells in the decidua and in the periphery from untreated control and RU486 treated pregnant mice. We observed a significant increase in the frequency of decidual Gal-9+ Th cells obtained from RU486 treated mice when compared to the periphery of treated mice or to the control untreated decidua (Fig 3B).

### TIM-3 expression by decidual and peripheral mononuclear cells from untreated control and RU486 treated pregnant mice

Next, we analyzed, how the expression of TIM-3 receptor is changed by NK, NKT, γ/δT and CD4+T cells at the materno-fetal interface as well as in the periphery of untreated control and Mifepristone treated pregnant mice.

**Fig 4:**
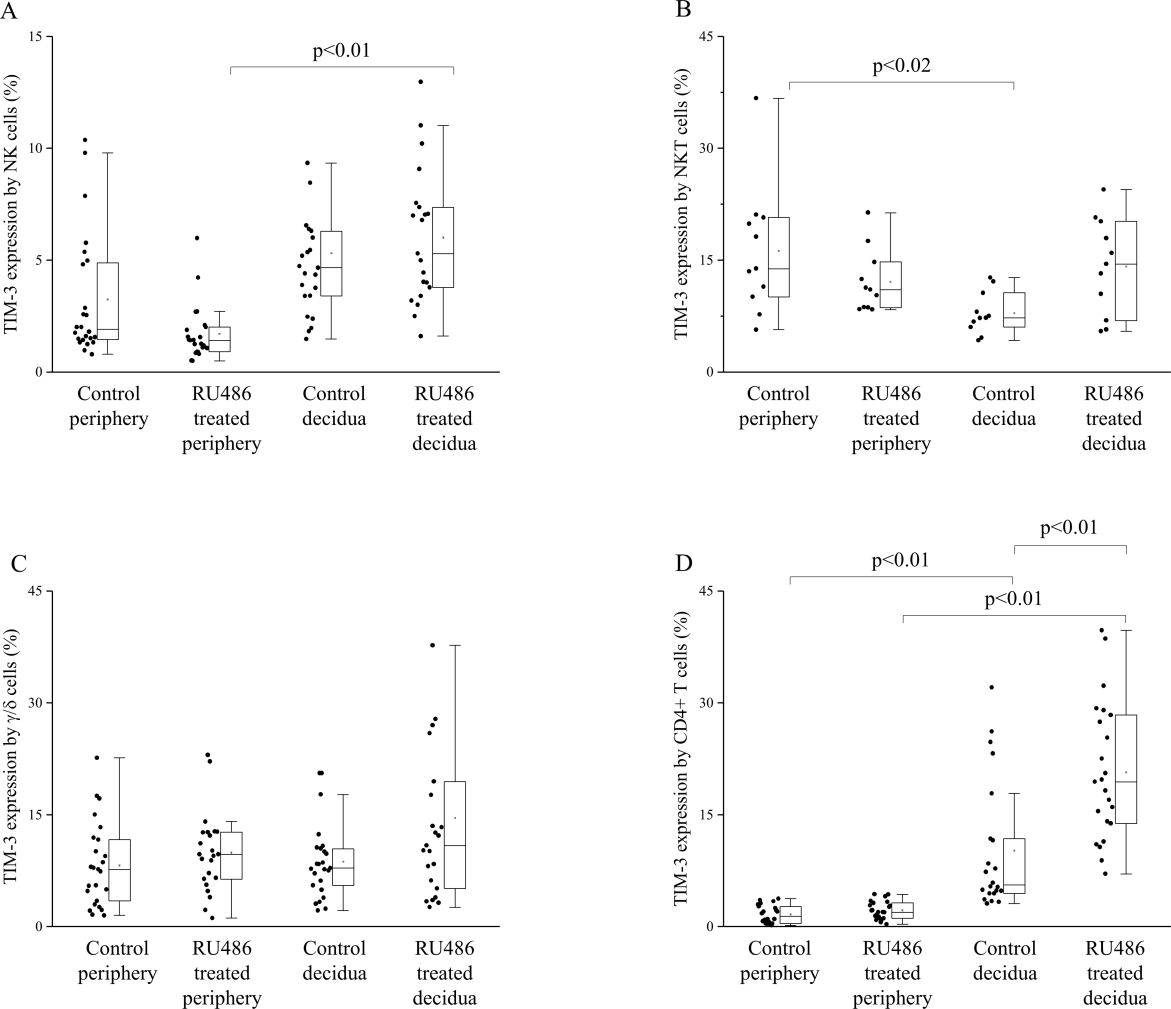
TIM-3 expression by decidual and peripheral mononuclear cells from untreated control and RU486 treated pregnant mice. Box plot of the median, the 25th and 75th percentiles, range, and individual data values for the expression of TIM-3 by NK cells, NKT cells, γ/δT and CD4 T cells in periphery and decidua in untreated and RU486 treated pregnant mice. The middle line within the box represents the median, the middle dot within the box represents the mean, the boxes indicate the interquartile ranges and the whiskers show the most extreme observations. Differences were considered statistically significant for p-values ≤0.05.

We found that both in control and RU486 treated mice group TIM-3 expression by CD4+T cells was significantly increased in the decidua compared to the periphery (Fig 4D). Contrary, TIM-3 expression by decidual NKT cells of untreated mice was significantly decreased when compared to the periphery (Fig 4B).

Following RU486 administration TIM-3 receptor expression by CD4+T cells was significantly increased at the materno-fetal interface compared to untreated mice (Fig 4D). In RU486 treated mice TIM-3 expression by NK cells was significantly increased in the decidua compared to the periphery (Fig 4A).

### CD107a expression by NK, NKT and γ/δ T cells in the decidua and in the periphery from untreated control and RU486 treated pregnant mice

**Fig 5:**
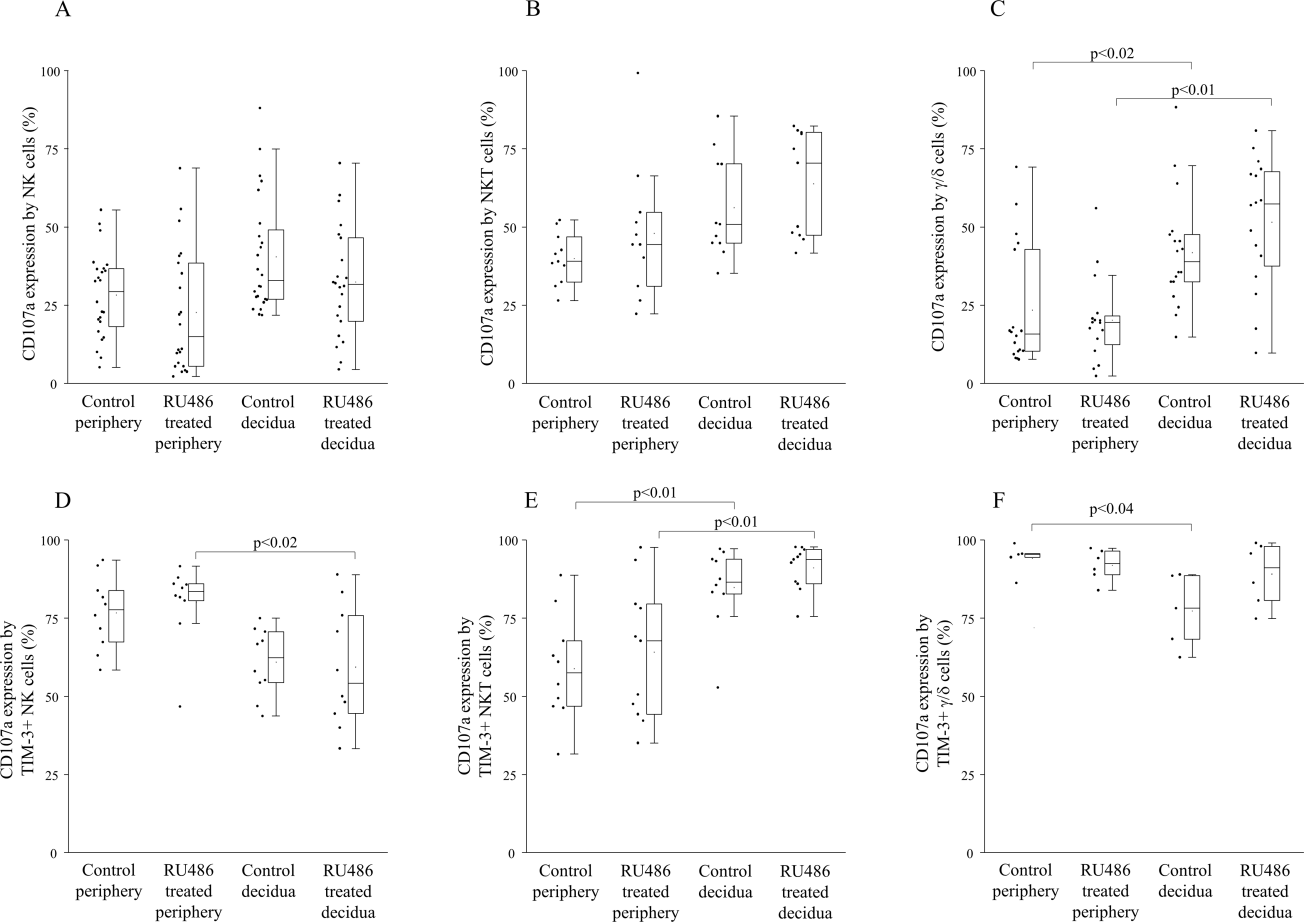
CD107a expression by different immune cell subsets and TIM-3 positive immune cell subsets in the periphery and in the decidua of untreated control and RU486 treated pregnant mice. Box plot of the median, the 25th and 75th percentiles, range, and individual data values for the he expression of CD107a by NK cells, NKT cells and γ/δT cells in the periphery and in the decidua of untreated control and RU486 treated pregnant mice (Fig 5A–5C). The expression of CD107a by TIM-3 positive NK cells, NKT cells and γ/δT cells in the periphery and in the decidua of untreated control and RU486 treated pregnant mice (Fig 5D–5F). The middle line within the box represents the median, the middle dot within the box represents the mean, the boxes indicate the interquartile ranges and the whiskers show the most extreme observations. Differences were considered statistically significant for p-values ≤0.05.

Investigating the expression of CD107a, which is known as a marker of cytotoxic potential by decidual immune cells obtained from the materno-fetal interface we found a significantly higher CD107a expression by decidual γ/δT cells compared to the periphery of untreated control mice (Fig 5C). In all other examined NK and T cell populations the CD107a positivity did not change.

In RU486 treated mice only decidual γ/δT cells showed significantly higher cytotoxic potential than their peripheral counterparts (Fig 5C).

In addition, we analyzed the CD107a expression within the TIM-3+ lymphocyte subsets. In untreated pregnant mice our results demonstrated a significantly decreased CD107a expression by decidual TIM-3+ γ/δT cells together with a significant increase in CD107a expression by NKT cells compared to the periphery (Fig 5F and 5E). Furthermore, in RU486 treated mice TIM-3+ decidual NKT cells showed significantly higher while NK cells showed significantly lower cytotoxic potential than their peripheral counterparts (Fig 5E and 5D).

## Discussion

Accumulating evidence has demonstrated that subpopulations of NK and T cells play a central role in the establishment and maintenance of materno-fetal immunotolerance and alteration in their physiological distribution or function might be associated with pregnancy complications. The exact mechanism underlying the fine regulation of these cells during Mifepristone (RU486) treatment is still under investigation.

Mifepristone has high affinity both for progesterone and glucocorticoid receptors. A number of studies suggest that Mifepristone may be involved in modulation of materno-fetal immune response. *In vitro* human experiments demonstrated that RU486 significantly inhibits the proliferation of lymphocytes [26], increases the cytotoxicity of peripheral NK cells [27] and blocks the immunosuppressive effect of progesterone mediated by Progesterone Induced Blocking Factor (PIBF) [28]. In addition it was shown that at a dose of 0.3–2 mg/kg RU486 could terminate early pregnancy in mice with an abortion rate of 60–100% [29,30] causing progesterone withdrawal, decreasing the number of PIBF expressing cells, enhancing the perforin expression of decidual NK cells and inducing increased peripheral NK activity [9,31]. On the other hand, late administration of Mifepristone (on gd 14–19) is able to induce preterm labor in mice at a dose of 0.4–12.5 mg/kg with a pregnancy termination rate of 66–100%.

Recent publications show that Gal-9/TIM-3 interaction plays an important role in the regulation of maternal immune tolerance and may be a potent regulator of the innate and adaptive immune response [12][20][32]. Therefore we aimed to investigate the role of Gal-9/Tim-3 pathway after low dose Mifepristone treatment using BALB/c syngeneic mouse pregnancy model at a gd of 14.5. Previously it was found that Gal-9 is widely expressed in the female reproductive tract at the feto-maternal interface [12,33–37] and by Treg or CD4+T (Th) cells [20][38]. It was also confirmed that Gal-9+Th cells are veritable immunosuppressors, which play a vital role in the regulation of Th17/Treg balance by producing IL-10/TGF-β [25].

Our previous human experiments revealed that serum Gal-9 levels and cell-surface expression of Gal-9 by peripheral CD4+T cells were increased throughout pregnancy [12]. Here we demonstrated that a relatively high proportion of peripheral NK, NKT and γ/δ T cells also showed Gal-9 positivity. Although Gal-9 expression by peripheral Treg cells was almost negligible, significantly higher percentage of Gal-9 positivity by decidual Treg cells was found in the decidua. Furthermore, we found that the expression of Gal-9 by NK cells was significantly decreased in the normal pregnant decidua compared to the periphery. 0.8mg/kg RU486-treatment resulted in a nearly complete disappearance of Gal-9 from the junctional zone of the placenta. Furthermore, the treatment significantly decreased the Gal-9 positivity of peripheral NK cells but significantly increased its expression by decidual Treg and CD4+T cells. In addition, the proportion of decidual Gal-9+Th cells with known suppressive capacity was significantly increased after Mifepristone administration.

Our data indicate that even a low dose Mifepristone treatment was effective enough to abrogate Gal-9 production of the placenta. The observed, increased Gal-9 expression by decidual Treg and CD4+Th cells suggest that local immunosuppressive mechanisms are also triggered 24 hours after the treatment, possibly to sustain impaired placental function. In addition, we suppose, that the accumulation of the Gal-9 secreting Gal-9+Th cells might be also involved to partly compensate the decreased placental Gal-9 expression at the materno-fetal interface. These mechanisms might inhibit the pro-inflammatory cytokine production of Th1 and Th17 cell by a Gal-9/TIM-3 dependent fashion [13] and aid the maintenance of the whole embryo-placenta unit.

On the basis of the above-mentioned results we hypothesize that beside the trophoblast and decidual cells, Gal-9+Th and Treg cells could serve as an alternative source of the locally secreted Gal-9 at the materno-fetal interface. During physiological conditions, the high local Gal-9 level could aid the development and suppressive function of Treg and Gal-9+Th cells, which might be involved in the inhibition of Th17 cell differentiation, downregulate the local Th1/Th17 cytokine production and induce apoptosis of Th1 CD4+T and activated CD8+T cells in the decidua. Additionally—to a lower extent—cytotoxic effector cells could be also able to produce this lectin both at the periphery and locally. We suggest that the membrane bound or secreted Gal-9 can engage the TIM-3 receptor on the surface of the immune effector cells and therefore it might directly or indirectly affect the innate and adaptive arm of the immune system at the materno-fetal interface.

After low dose Mifepristone treatment, the placental expression of Gal-9 might be seriously impaired. Since local Gal-9 level could be important for placental development and for the regulation of the maternal anti-fetal immune response, the disruption of Gal-9- regulated tolerance mechanisms can contribute to pregnancy complications [39] and lead to pregnancy termination or preterm labor. Harmful effects caused by low dose Mifepristone treatment could be physiologically compensated via the upregulation of Gal-9 expression by decidual Treg and CD4+Th cells and by the local accumulation and/or differentiation of Gal-9 secreting Treg and Gal-9+ Th cells. We suggest that these compensatory mechanisms might sustain impaired placental function when only partial progesterone withdrawal is induced, but might become ineffective when higher dose of Mifepristone would be used. Administration of 2 mg/kg or higher dose of RU486 can cause complete blocking of progesterone receptors causing estrogen dominance, sensitizing the uterus to the activity of the prostaglandin, resulting in intrauterine fetal death or a definite premature labor at a late stage (gd 14–19) of murine pregnancy.

Beside the above-mentioned actions, it is well established, that during pregnancy RU486 can induce an immunological alteration in a Gal-9/TIM-3 independent way. The regulation of Th1/Th2/Th17/Treg paradigm is one of the most important mechanisms by which progesterone exerts its pregnancy-protective effect [40]. Progesterone induces a Th1 to Th2-type immunity skew by inducing the expression of PIBF and Th2-type cytokines and suppressing the production of Th1 cytokines [41]. As a receptor antagonist, Mifepristone can inhibit the pregnancy-protective immunological effects of progesterone in a TIM-3 independent way by inhibiting the proliferation of lymphocytes [26] and inducing the production of prostaglandins, cyclooxygenases and proinflammatory cytokines *in vitro*. These effects—together with the dysregulated Gal-9/TIM-3 pathway—might alter the local cytokine milieu from an immunosuppressive/angiogenic environment towards a proinflammatory condition. This can disturb the precise regulation of the Th1/Th2/Th17/Treg balance and induce the activation of local inflammatory and cytotoxic immune response leading to pregnancy loss [42][43][44].

The limitations of our study is that (1) we used only one dose of Mifepristone (0.8 mg/kg) instead of a several different doses from low to high, (2) we tested the in vivo effect of RU486 only on gestational day 14.5 but not earlier (3) and analysis was performed just 24h after treatment. Based on the literature higher doses of Mifepristone (e.g. >2 mg/kg) would cause complete abortion after 48h as indicated in a paper by Lv et al.[30]. In our case these setting were not applicable since after complete abortion tissue destruction strongly limit to obtain enough decidual lymphocytes for flow cytometric analyses.

## Supporting information

S1 Fig Gating strategy to detect peripheral immune cell populations. Shows the gating technique used to detect immune cell populations in the periphery.
(TIF)

S2 Fig Gating strategy to detect decidual immune cell populations. Shows the gating technique used to detect decidual immune cell populations. CD45+ leukocytes were gated using SSC and FL-5 (PerCP) parameters. Endothelial cells which may fall inside the lymhogate were excluded by CD45 staining. Lymphogate was created based on physical characteristics typical of lymphoid cells using forward and side scatter parameters. All further analyses of decidual immune cells were performed on CD45+ cells only, by combining the lymphogate and CD45+ cell gate.
(TIF)

S3 Fig Representative histograms showing TIM-3, Galectin-9 and isotype control staining. Representative histograms showing TIM-3, Galectin-9 and isotype control staining.
(TIF)

S4 Fig Representative dot plots showing TIM-3 staining by decidual and peripheral mononuclear cells from untreated control and RU486 treated pregnant mice. Representative dot plots showing TIM-3 expression by NK cells, NKT cells, γ/δT and CD4 T cells in periphery and decidua of untreated and RU486 treated pregnant mice.
(TIF)

S5 Fig Representative dot plots showing Gal-9 staining by decidual and peripheral mononuclear cells from untreated control and RU486 treated pregnant mice. Representative dot plots showing Gal-9 expression by NK cells, NKT cells, γ/δT and Treg cells in periphery and decidua of untreated and RU486 treated pregnant mice.
(TIF)

## References

[1] Sedgh G, Bearak J, Singh S, Bankole A, Popinchalk A, Ganatra B, et al Abortion incidence between 1990 and 2014: global, regional, and subregional levels and trends. Lancet (London, England). 2016;388: 258–67. doi: 10.1016/S0140-6736(16)30380-4 10.1016/S0140-6736(16)30380-4 PMC5498988 27179755

[2] Kapp N, Whyte P, Tang J, Jackson E, Brahmi D. A review of evidence for safe abortion care. Contraception. 2013;88: 350–63. doi: 10.1016/j.contraception.2012.10.027 23261233 10.1016/j.contraception.2012.10.027

[3] Nissi R, Santala M, Immonen E, Talvensaari-Mattila A. Mifepristone and misoprostol is safe and effective method in the second-trimester pregnancy termination. Arch Gynecol Obstet. 2016;294: 1243–1247. doi: 10.1007/s00404-016-4169-8 27522599 10.1007/s00404-016-4169-8

[4] Louie KS, Chong E, Tsereteli T, Avagyan G, Abrahamyan R, Winikoff B. Second trimester medical abortion with mifepristone followed by unlimited dosing of buccal misoprostol in Armenia. Eur J Contracept Reprod Heal Care. 2017;22: 76–80. doi: 10.1080/13625187.2016.1258461 10.1080/13625187.2016.1258461 27871191

[5] Raymond EG, Shannon C, Weaver MA, Winikoff B. First-trimester medical abortion with mifepristone 200 mg and misoprostol: a systematic review. Contraception. 2013;87: 26–37. doi: 10.1016/j.contraception.2012.06.011 22898359 10.1016/j.contraception.2012.06.011

[6] Mao G, Wang J, Kang Y, Tai P, Wen J, Zou Q, et al Progesterone increases systemic and local uterine proportions of CD4+CD25+ Treg cells during midterm pregnancy in mice. Endocrinology. 2010;151: 5477–88. doi: 10.1210/en.2010-0426 20844003 10.1210/en.2010-0426

[7] Li X, Wang B, Li Y, Wang L, Zhao X, Zhou X, et al The Th1/Th2/Th17/Treg paradigm induced by stachydrine hydrochloride reduces uterine bleeding in RU486-induced abortion mice. J Ethnopharmacol. 2013;145: 241–253. doi: 10.1016/j.jep.2012.10.059 23178269 10.1016/j.jep.2012.10.059

[8] Zhong X, Shi W, Ma A, Wang X, Zhang J, Li X. Influence of Radix scutellariae on Th1/Th2 cytokine balance in RU486-induced abortion in mice. Front Agric China. Higher Education Press; 2007;1: 96–100. doi: 10.1007/s11703-007-0018-7

[9] Bogdan A, Berta G, Szekeres-Bartho J. PIBF positive uterine NK cells in the mouse decidua. J Reprod Immunol. 2017;119: 38–43. doi: 10.1016/j.jri.2016.12.001 28043035 10.1016/j.jri.2016.12.001

[10] Boenisch IG, Najafian N, Akiba H, Yagita H, Zhu I, Batal PJ, et al Induce Fetomaternal Tolerance TIM-3 Regulates Innate Immune Cells To TIM-3 Regulates Innate Immune Cells To Induce Fetomaternal Tolerance. J Immunol Mater Suppl J Immunol by guest. 2013;190: 88–96. doi: 10.4049/jimmunol.1202176 10.4049/jimmunol.1202176 PMC3529820 23180822

[11] Sánchez-Fueyo A, Tian J, Picarella D, Domenig C, Zheng XX, Sabatos CA, et al Tim-3 inhibits T helper type 1-mediated auto- and alloimmune responses and promotes immunological tolerance. Nat Immunol. 2003;4: 1093–101. doi: 10.1038/ni987 14556005 10.1038/ni987

[12] Meggyes M, Miko E, Polgar B, Bogar B, Farkas B, Illes Z, et al Peripheral blood TIM-3 positive NK and CD8+ T cells throughout pregnancy: TIM-3/galectin-9 interaction and its possible role during pregnancy. PLoS One. 2014;9: e92371 doi: 10.1371/journal.pone.0092371 24651720 10.1371/journal.pone.0092371 PMC3961322

[13] Hastings WD, Anderson DE, Kassam N, Koguchi K, Greenfield EA, Kent SC, et al TIM-3 is expressed on activated human CD4+ T cells and regulates Th1 and Th17 cytokines. Eur J Immunol. 2009;39: 2492–501. doi: 10.1002/eji.200939274 19676072 10.1002/eji.200939274 PMC2759376

[14] Zhao J, Lei Z, Liu Y, Li B, Zhang L, Fang H, et al Human pregnancy up-regulates Tim-3 in innate immune cells for systemic immunity. J Immunol. 2009;182: 6618–24. doi: 10.4049/jimmunol.0803876 19414817 10.4049/jimmunol.0803876

[15] van de Weyer PS, Muehlfeit M, Klose C, Bonventre J V, Walz G, Kuehn EW. A highly conserved tyrosine of Tim-3 is phosphorylated upon stimulation by its ligand galectin-9. Biochem Biophys Res Commun. 2006;351: 571–6. doi: 10.1016/j.bbrc.2006.10.079 17069754 10.1016/j.bbrc.2006.10.079

[16] Lee J, Su EW, Zhu C, Hainline S, Phuah J, Moroco JA, et al Phosphotyrosine-dependent coupling of Tim-3 to T-cell receptor signaling pathways. Mol Cell Biol. 2011;31: 3963–74. doi: 10.1128/MCB.05297-11 21807895 10.1128/MCB.05297-11 PMC3187355

[17] Seki M, Oomizu S, Sakata K-MM, Sakata A, Arikawa T, Watanabe K, et al Galectin-9 suppresses the generation of Th17, promotes the induction of regulatory T cells, and regulates experimental autoimmune arthritis. Clin Immunol. 2008;127: 78–88. doi: 10.1016/j.clim.2008.01.006 18282810 10.1016/j.clim.2008.01.006

[18] Zhu C, Anderson AC, Schubart A, Xiong H, Imitola J, Khoury SJ, et al The Tim-3 ligand galectin-9 negatively regulates T helper type 1 immunity. Nat Immunol. 2005;6: 1245–52. doi: 10.1038/ni1271 16286920 10.1038/ni1271

[19] Sabatos CA, Chakravarti S, Cha E, Schubart A, Sánchez-Fueyo A, Zheng XX, et al Interaction of Tim-3 and Tim-3 ligand regulates T helper type 1 responses and induction of peripheral tolerance. Nat Immunol. 2003;4: 1102–10. doi: 10.1038/ni988 14556006 10.1038/ni988

[20] Meggyes M, Lajko A, Palkovics T, Totsimon A, Illes Z, Szereday L, et al Feto-maternal immune regulation by TIM-3/galectin-9 pathway and PD-1 molecule in mice at day 14.5 of pregnancy. Placenta. 2015;36: 1153–60. doi: 10.1016/j.placenta.2015.07.124 26278059 10.1016/j.placenta.2015.07.124

[21] Alter G, Malenfant JM, Altfeld M. CD107a as a functional marker for the identification of natural killer cell activity. J Immunol Methods. 2004;294: 15–22. doi: 10.1016/j.jim.2004.08.008 15604012 10.1016/j.jim.2004.08.008

[22] Lu S, Wu R, Wang Z. Expression of T-lymphocytes and cytokines in the decidua of mifepristone with misoprostol for terminating early pregnancy. [Chinese]. Zhonghua Fu Chan Ke Za Zhi. 2001;36: 625–627. Available: http://www.ncbi.nlm.nih.gov/pubmed/16134529 16134529

[23] Chen Y, Wang Y, Zhuang Y, Zhou F, Huang L. Mifepristone increases the cytotoxicity of uterine natural killer cells by acting as a glucocorticoid antagonist via ERK activation. PLoS One. 2012;7: e36413 doi: 10.1371/journal.pone.0036413 22563497 10.1371/journal.pone.0036413 PMC3341349

[24] Milne SA, Henderson TA, Kelly RW, Saunders PT, Baird DT, Critchley HOD. Leukocyte populations and steroid receptor expression in human first-trimester decidua; regulation by antiprogestin and prostaglandin E analog. J Clin Endocrinol Metab. 2005;90: 4315–21. doi: 10.1210/jc.2004-2338 15814773 10.1210/jc.2004-2338

[25] Oomizu S, Arikawa T, Niki T, Kadowaki T, Ueno M, Nishi N, et al Cell surface galectin-9 expressing th cells regulate Th17 and Foxp3+ Treg development by galectin-9 secretion. PLoS One. 2012;7: e48574 doi: 10.1371/journal.pone.0048574 23144904 10.1371/journal.pone.0048574 PMC3492452

[26] Chien CH, Lai JN, Liao CF, Wang OY, Lu LM, Huang MI, et al Mifepristone acts as progesterone antagonist of non-genomic responses but inhibits phytohemagglutinin-induced proliferation in human T cells. Hum Reprod. 2009;24: 1968–75. doi: 10.1093/humrep/dep099 19401324 10.1093/humrep/dep099

[27] Hansen KA, Opsahl MS, Nieman LK, Baker JR, Klein TA. Natural killer cell activity from pregnant subjects is modulated by RU 486. Am J Obstet Gynecol. 1992;166: 87–90. Available: http://www.ncbi.nlm.nih.gov/pubmed/1733224 1733224 10.1016/0002-9378(92)91835-x

[28] Szekeres-Bartho J, Chaouat G, Kinsky R. A progesterone-induced blocking factor corrects high resorption rates in mice treated with antiprogesterone. Am J Obstet Gynecol. 1990;163: 1320–2. Available: http://www.ncbi.nlm.nih.gov/pubmed/2220944 2220944 10.1016/0002-9378(90)90713-h

[29] Szekeres-Bartho J, Par G, Dombay G, Smart YC, Volgyi Z. The Antiabortive Effect of Progesterone-Induced Blocking Factor in Mice Is Manifested by Modulating NK Activity. Cell Immunol. 1997;177: 194–199. doi: 10.1006/cimm.1997.1090 9178647 10.1006/cimm.1997.1090

[30] Lv F, Xu X, Zhang S, Wang L, Wang N, He B, et al Repeated abortion affects subsequent pregnancy outcomes in BALB/c mice. PLoS One. Public Library of Science; 2012;7: e48384 doi: 10.1371/journal.pone.0048384 23119001 10.1371/journal.pone.0048384 PMC3485242

[31] Szekeres-Bartho J, Polgar B, Kozma N, Miko E, Par G, Szereday L, et al Progesterone-dependent immunomodulation. Chem Immunol Allergy. 2005;89: 118–25. doi: 10.1159/000087953 16129958 10.1159/000087953

[32] Hu X-H, Tang M-X, Mor G, Liao A-H. Tim-3: Expression on immune cells and roles at the maternal-fetal interface. J Reprod Immunol. 2016;118: 92–99. doi: 10.1016/j.jri.2016.10.113 27792886 10.1016/j.jri.2016.10.113

[33] Popovici RM, Krause MS, Germeyer A, Strowitzki T, Von Wolff M. Galectin-9: A new endometrial epithelial marker for the mid- and late-secretory and decidual phases in humans. J Clin Endocrinol Metab. 2005;90: 6170–6176. doi: 10.1210/jc.2004-2529 16105962 10.1210/jc.2004-2529

[34] Atsukawa M, Nakatsuka K, Kobayashi T, Shimizu M, Tamura H, Harimoto H, et al Ribavirin downmodulates inducible costimulator on CD4+ T cells and their interleukin-10 secretion to assist in hepatitis C virus clearance. J Gastroenterol Hepatol. 2012;27: 823–31. doi: 10.1111/j.1440-1746.2011.06882.x 21871023 10.1111/j.1440-1746.2011.06882.x

[35] Shimizu Y, Kabir-Salmani M, Azadbakht M, Sugihara K, Sakai K, Iwashita M. Expression and localization of galectin-9 in the human uterodome. Endocr J. 2008;55: 879–87. Available: http://www.ncbi.nlm.nih.gov/pubmed/18506087 18506087 10.1507/endocrj.k08e-111

[36] Than NG, Romero R, Goodman M, Weckle A, Xing J, Dong Z, et al A primate subfamily of galectins expressed at the maternal-fetal interface that promote immune cell death. Proc Natl Acad Sci U S A. 2009;106 doi: 10.1073/pnas.0903568106 10.1073/pnas.0903568106 PMC2689813 19497882

[37] Li Y-H, Zhou W-H, Tao Y, Wang S-C, Jiang Y-L, Zhang D, et al The Galectin-9/Tim-3 pathway is involved in the regulation of NK cell function at the maternal-fetal interface in early pregnancy. Cell Mol Immunol. 2015; doi: 10.1038/cmi.2014.126 10.1038/cmi.2014.126 PMC4711677 25578313

[38] Sun J, Yang M, Ban Y, Gao W, Song B, Wang Y, et al Tim-3 Is Upregulated in NK Cells during Early Pregnancy and Inhibits NK Cytotoxicity toward Trophoblast in Galectin-9 Dependent Pathway. PLoS One. 2016;11: e0147186 doi: 10.1371/journal.pone.0147186 26789128 10.1371/journal.pone.0147186 PMC4720443

[39] Heusschen R, Freitag N, Tirado-González I, Barrientos G, Moschansky P, Muñoz-Fernández R, et al Profiling Lgals9 splice variant expression at the fetal-maternal interface: implications in normal and pathological human pregnancy. Biol Reprod. 2013;88: 22 doi: 10.1095/biolreprod.112.105460 23242525 10.1095/biolreprod.112.105460

[40] Lee JH, Ulrich B, Cho J, Park J, Kim CH. Progesterone promotes differentiation of human cord blood fetal T cells into T regulatory cells but suppresses their differentiation into Th17 cells. J Immunol. 2011;187: 1778–87. doi: 10.4049/jimmunol.1003919 21768398 10.4049/jimmunol.1003919 PMC3155957

[41] Miyaura H, Iwata M. Direct and indirect inhibition of Th1 development by progesterone and glucocorticoids. J Immunol. 2002;168: 1087–94. Available: http://www.ncbi.nlm.nih.gov/pubmed/11801642 11801642 10.4049/jimmunol.168.3.1087

[42] Piccinni M-P. T cell tolerance towards the fetal allograft. J Reprod Immunol. 2010;85: 71–75. doi: 10.1016/j.jri.2010.01.006 20334928 10.1016/j.jri.2010.01.006

[43] Liu Y-S, Wu L, Tong X-H, Wu L-M, He G-P, Zhou G-X, et al Study on the relationship between Th17 cells and unexplained recurrent spontaneous abortion. Am J Reprod Immunol. 2011;65: 503–11. doi: 10.1111/j.1600-0897.2010.00921.x 21029245 10.1111/j.1600-0897.2010.00921.x

[44] Saito S, Nakashima A, Shima T, Ito M. Th1/Th2/Th17 and regulatory T-cell paradigm in pregnancy. Am J Reprod Immunol. 2010;63: 601–10. doi: 10.1111/j.1600-0897.2010.00852.x 20455873 10.1111/j.1600-0897.2010.00852.x

