# Supplementary figures and images for: The immunological effect of Galectin-9/TIM-3 pathway after low dose Mifepristone treatment in mice at 14.5 day of pregnancy
Source: PLoS One. 2018 Mar 22;13(3):e0194870. doi: 10.1371/journal.pone.0194870 (PMC5864070; doi:10.1371/journal.pone.0194870)

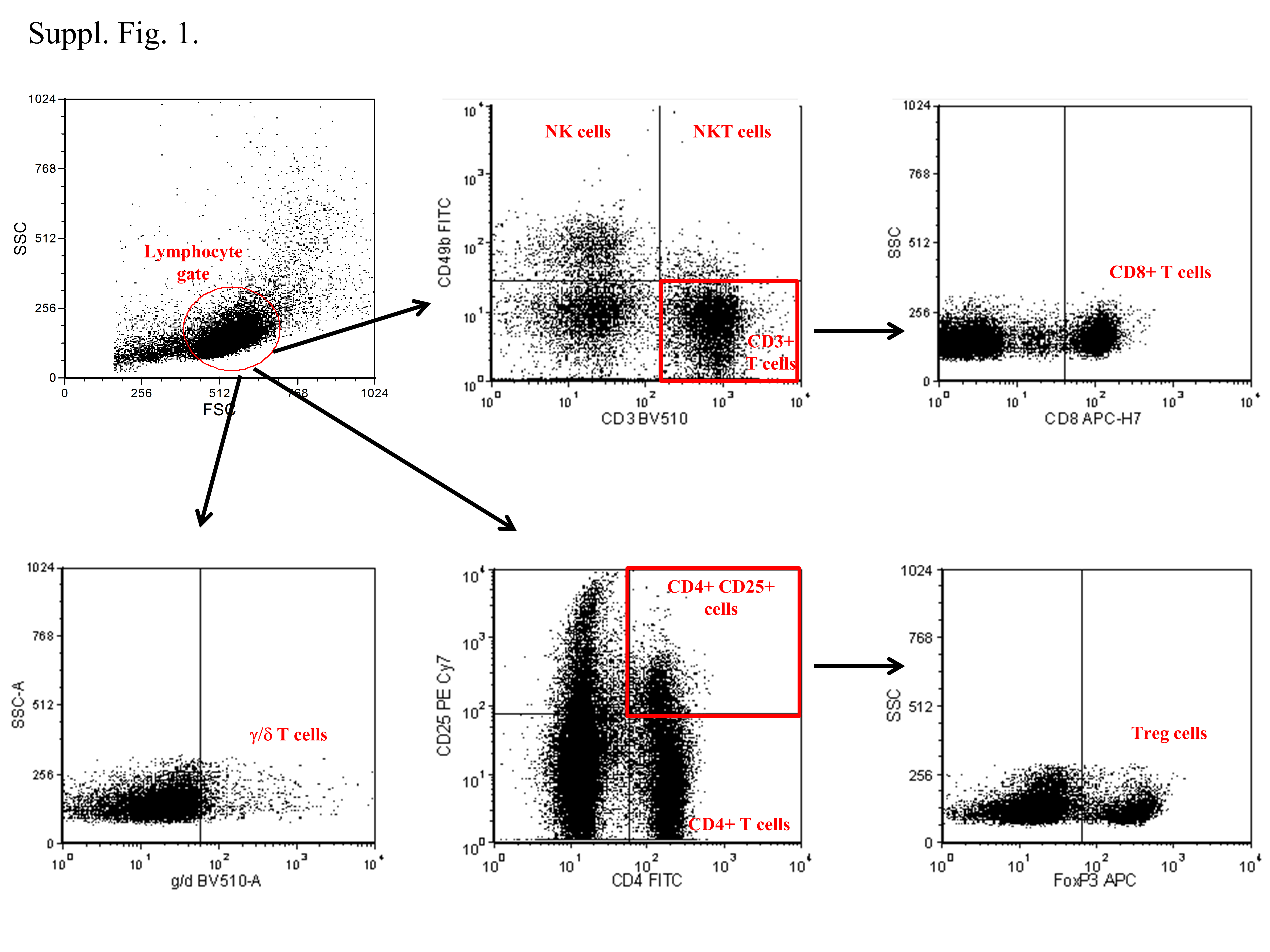

Supplement: S1 Fig — Shows the gating technique used to detect immune cell populations in the periphery. (TIF) [file pone.0194870.s001.TIF]

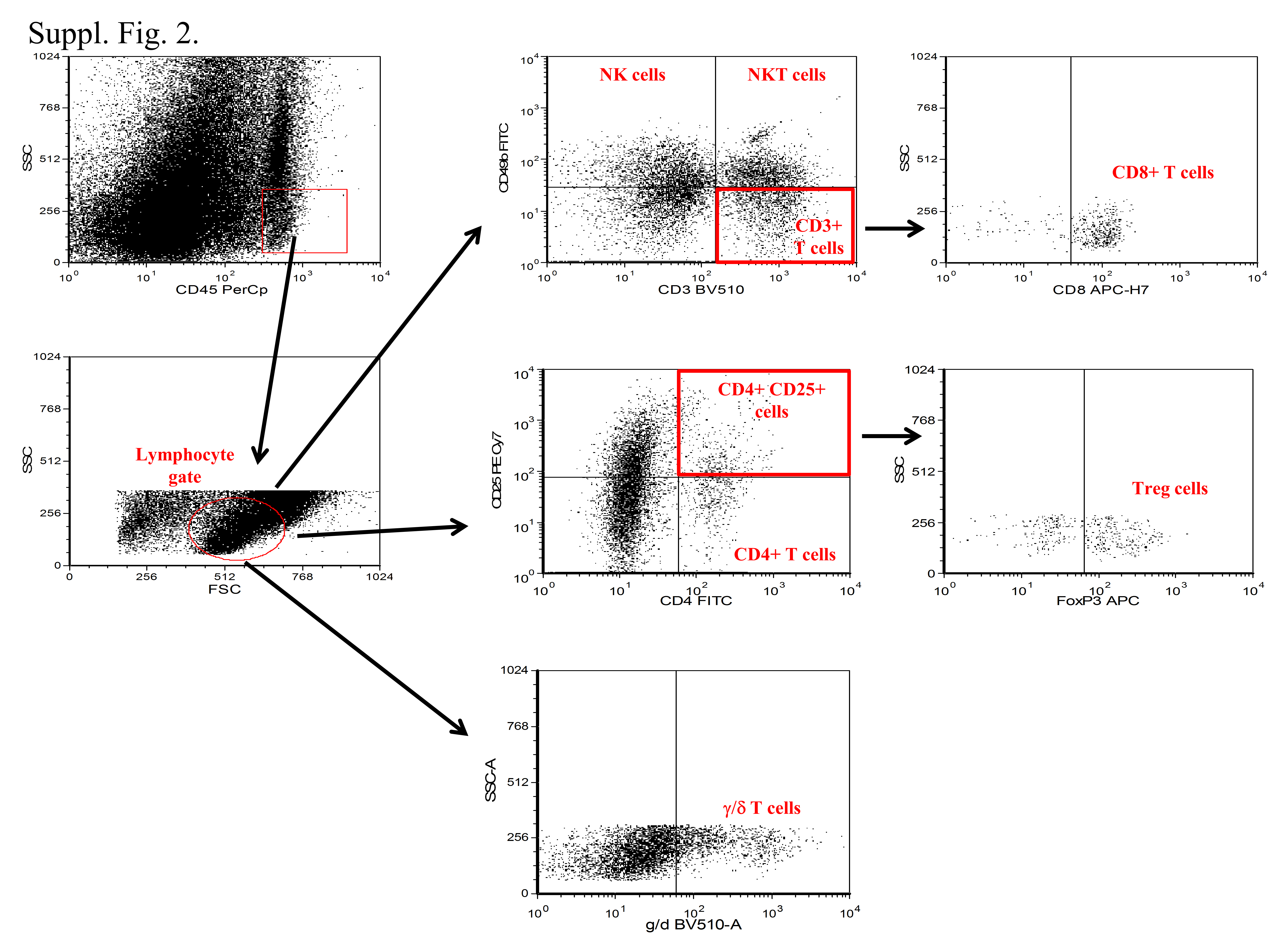

Supplement: S2 Fig — Shows the gating technique used to detect decidual immune cell populations. CD45+ leukocytes were gated using SSC and FL-5 (PerCP) parameters. Endothelial cells which may fall inside the lymhogate were excluded by CD45 staining. Lymphogate was created based on physical characteristics typical of lymphoid cells using forward and side scatter parameters. All further analyses of decidual immune cells were performed on CD45+ cells only, by combining the lymphogate and CD45+ cell gate. (TIF) [file pone.0194870.s002.TIF]

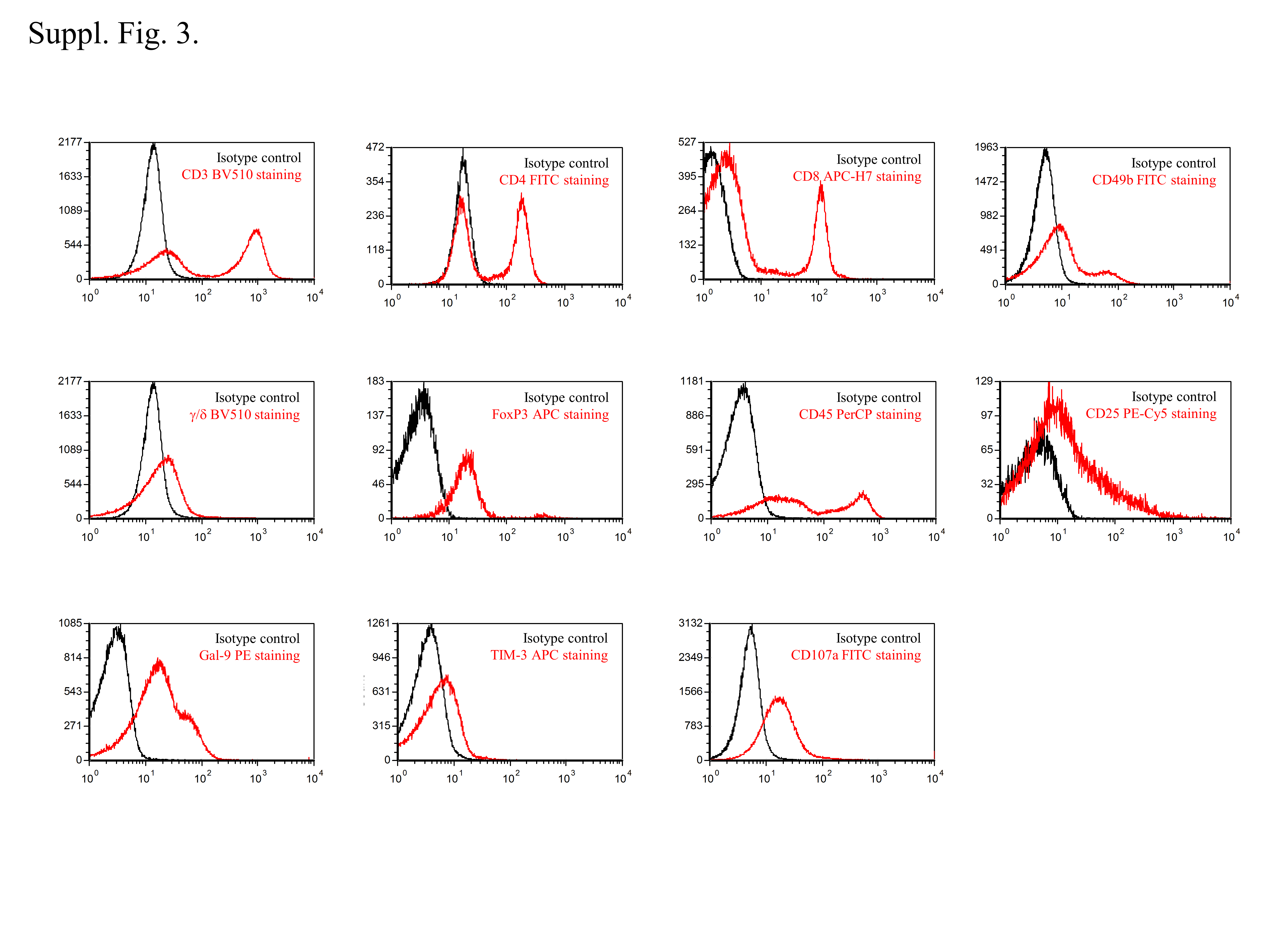

Supplement: S3 Fig — Representative histograms showing TIM-3, Galectin-9 and isotype control staining. (TIF) [file pone.0194870.s003.TIF]

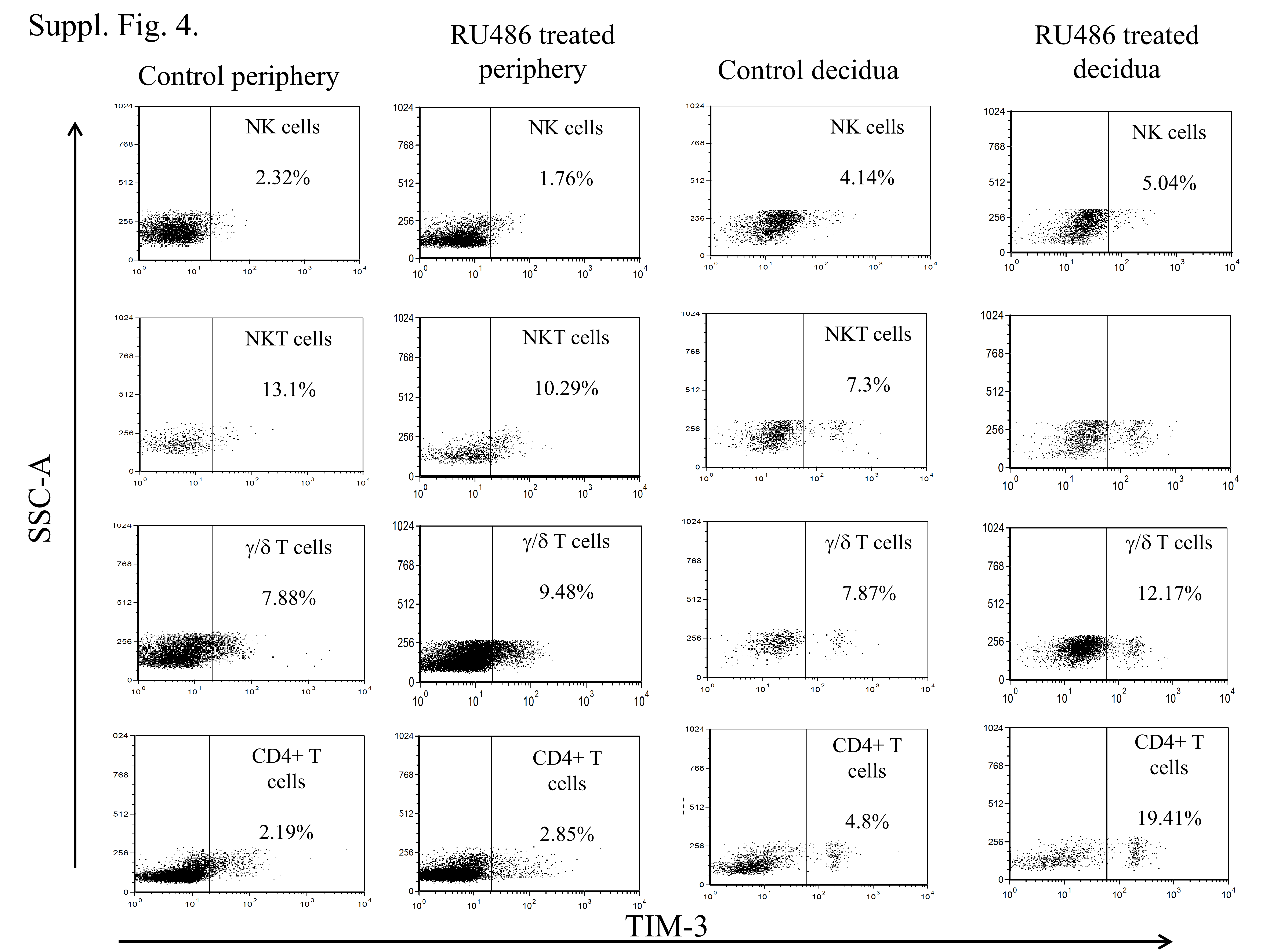

Supplement: S4 Fig — Representative dot plots showing TIM-3 expression by NK cells, NKT cells, γ/δT and CD4 T cells in periphery and decidua of untreated and RU486 treated pregnant mice. (TIF) [file pone.0194870.s004.TIF]

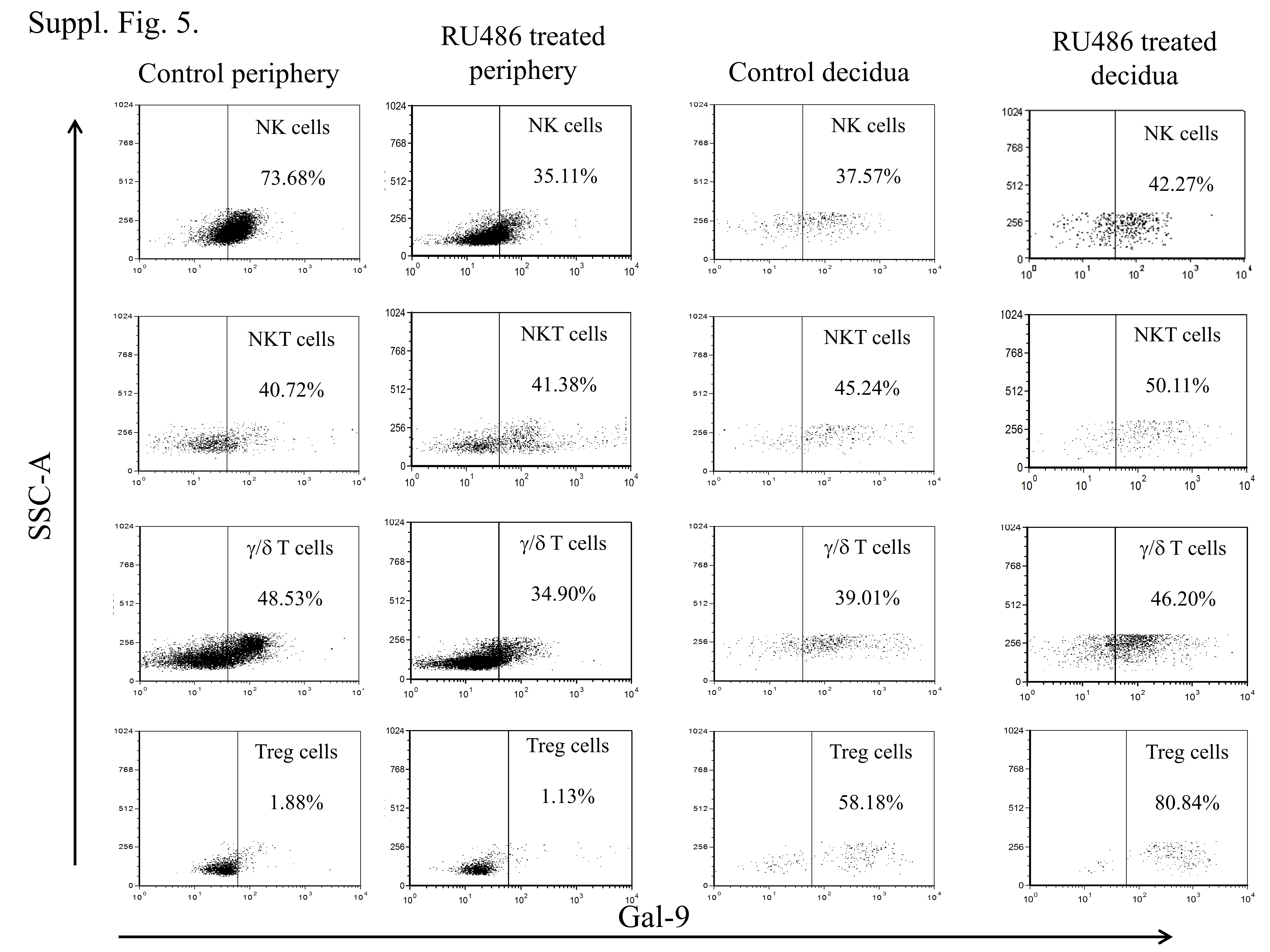

Supplement: S5 Fig — Representative dot plots showing Gal-9 expression by NK cells, NKT cells, γ/δT and Treg cells in periphery and decidua of untreated and RU486 treated pregnant mice. (TIF) [file pone.0194870.s005.TIF]
